# Adding Far-Red to Red, Blue Supplemental Light-Emitting Diode Interlighting Improved Sweet Pepper Yield but Attenuated Carotenoid Content

**DOI:** 10.3389/fpls.2022.938199

**Published:** 2022-06-21

**Authors:** Dongpil Kim, Jung Eek Son

**Affiliations:** ^1^Department of Agriculture, Forestry and Bioresources, Seoul National University, Seoul, South Korea; ^2^Research Institute of Agriculture and Life Sciences, Seoul National University, Seoul, South Korea

**Keywords:** far-red fraction, fruit color, light environment, soluble sugar, supplemental lighting

## Abstract

Supplemental interlighting is commonly used in modern greenhouses to improve light deficiency, but the light spectrum affects fruit quality and color change. This study aimed to analyze the effect of interlighting with red, blue, and additional far-red light on the fruit qualities and carotenoid contents of red and yellow sweet peppers (*Capsicum annuum* L.). Three light treatments were applied: natural light (NL), NL with red + blue LED interlighting (71 μmol m^–2^ s^–1^) (RB), and RB with far-red light (55 μmol m^–2^ s^–1^) (RBFR). Ascorbic acid, free sugars, and individual carotenoid content were quantified with HPLC analysis. Fruits were sampled on 2020.11.14 (Group 1) and 2021.01.03 (Group 2) from the plants grown under average light intensities of 335.9 and 105.6 μmol m^–2^ s^–1^, respectively. In the overall period, total yields in RB and RBFR were 22 and 33% higher than those in NL in red fruits and 2 and 21% higher in yellow fruits, respectively. In both colored fruits, ascorbic acid, total soluble sugar, and carotenoid content were higher in RB and RBFR than NL. In Group 1, ascorbic acid and total soluble sugar were significantly different between RB and RBFR only in red fruits. In Group 2, ascorbic acids in red and yellow fruits were 9 and 3% higher in RBFR than RB but total soluble sugars were 4 and 2% lower, respectively. Carotenoid contents in red and yellow fruits were 3.0- and 2.1-fold higher in RB and 2.0- and 1.4-fold higher in RBFR than those in NL, respectively. In this study, interlighting had a significant impact on fruit quality in Group 2, mainly due to the increase in the ratio of interlighting to total light by seasonal changes. In particular, red and yellow fruit yields were 9% and 19% higher in RBFR than RB, but carotenoid contents were 26 to 9% lower, respectively. This result exhibited that additional far-red lighting has a trade-off relationship between fruit yield and carotenoid content. Thus, it is necessary to provide an adequate light spectrum according to a specific cultivation purpose, such as improving yield or accumulating plastids in fruits.

## Introduction

Sweet pepper is one of the most-consumed vegetables worldwide due to its taste and higher nutrient values, such as vitamin C and carotenoid content (Kim et al., 2011). In addition to the sensory attributes, health properties, such as vitamin C and carotenoids, also emerge as factors that determine the quality of sweet pepper. Carotenoids determine the color of the fruit surface and function as antioxidants for scavenging free radicals that help protect cells from oxidation damage. In terms of the human diet, carotenoids are essential and are broken down into provitamin A in the body to improve eye health and decrease the risk of cardiovascular disease and cancers (Maoka et al., 2001; Abdel-Aal et al., 2013).

In the northern hemisphere, sweet peppers are mainly grown in greenhouses due to their perennial characteristics in tropical areas. Therefore, light is a limiting factor for fruit yields and quality. As a countermeasure, supplemental lighting is generally used to produce high-quality fruits year-round under the insufficient amounts of sunlight in modern greenhouses. Among various light sources, light-emitting diodes (LEDs) have the advantage of controlling the spectrum of light sources and thereby enable the photophysiological induction of crops. Red and blue wavelengths are representative light spectra for higher quantum yields and photosynthetic efficiencies (McCree, 1971). In addition to red and blue light, far-red (FR) light is also being introduced as a light source that mediates the photoreceptor responses from plant phytochromes (Mitchell, 2015). Regarding the effect of FR light, several studies have been conducted on tomatoes (Ji et al., 2019; Kalaitzoglou et al., 2019; Palmitessa et al., 2020). Adding FR to red light improved the chemical compositions, such as fruit sugar contents and mineral components, and increased the fruit yields of greenhouse tomatoes (Kim et al., 2020). However, studies on fruit yields and quality in sweet peppers under red, blue, or additional FR light are not yet sufficient.

Phytochromes are homodimeric photoreceptors that shift between two equilibrium forms in plants: the biologically inactive form P_*r*_ has a maximum absorbance in red light (660 nm), and the biologically active P_*fr*_ form has a maximum absorbance in FR light (730 nm) and is excited by absorbing red light (Sharrock, 2008). Activated P_*fr*_ migrates from the cytosol to the nucleus and exerts various biological responses by selective binding to transcription factors, such as those in the phytochrome-interacting factor (PIF) family (Casal, 2013). In tomatoes or sweet peppers, fruit ripening simultaneously proceeds during chlorophyll breakdown and accumulation of carotenoids (Seymour et al., 2013). The chlorophylls in immature fruit surfaces generate a self-shading effect due to preferential absorption of red light that maintains the phytochromes predominantly in the inactive P_*r*_ form and high PIF1a, levels of PIF homolog that suppress *PSY1* expression, which is the first enzyme responsible for carotenoid metabolism (Llorente et al., 2016). Thus, under low R:FR, carotenoid production could be prevented by inhibiting the genes involved in the carotenoid biosynthesis pathway by activating PIFs. Against this background, a high proportion of FR light can act as a trade-off for ripening progress or carotenoid production by maintaining a relatively higher equilibrium of inactive P_*r*_.

In previous studies, the addition of photosynthetically active radiation (PAR) *via* LEDs resulted in similar responses at higher R:FR ratios. Monochromatic red, blue, and white LED lighting was efficacious in increasing lycopene and lutein production in tomatoes (Dannehl et al., 2021). In addition, the lycopene and capsanthin syntheses of tomatoes and peppers have been enhanced under supplementary red or blue light LEDs with higher expression of carotenoid biosynthesis genes, such as the *Psy*, *Lcyb*, and *Ccs* genes (Pola et al., 2019; Xie et al., 2019). These results supported that supplemental lighting with red and blue LEDs could result in higher carotenoid content in sweet peppers, but the effect of additional far-red light is unclear. To our knowledge, studies on fruit carotenoid accumulation by adding FR light to red and blue interlighting have rarely been conducted under greenhouse conditions. Therefore, it is necessary to analyze whether interlighting with additional FR light affects fruit quality in greenhouses with background sunlight.

The purpose of this study was to analyze the effect of interlighting with red, blue, and additional FR lighting on the fruit yields, quality, and carotenoid accumulation of sweet pepper fruits in greenhouses.

## Materials and Methods

### Plant Materials and Growth Conditions

Sweet pepper seeds of red (Mavera, Enza Zaden, North Holland, Netherlands) and yellow (Florate, Enza Zaden) fruit colors were sown into soilless plugs with sizes of 2.0 W × 2.0 L × 2.7 H cm each (Plantop Plug, Grodan, Roermond, Netherlands), and the plug trays were covered with vermiculites to retain moisture during germination. When two cotyledons were fully developed, the plug seedlings were transferred into stonewool blocks (Grodan Plantop, Grodan) and germinated in a commercial nursery greenhouse at Asan, South Korea (36.8°N, 127.1°E). During the seedling period, a nutrient solution of EC 1.2 mS cm^–1^ was supplied with soluble NPK (3:1:1) fertilizer (Multifeed, Haifa Group, Haifa, Israel). After a 6-week nursery period, the seedlings were transplanted into stonewool substrates (Grodan GT Master, Grodan) on 2020.08.26, in a Venlo-type glasshouse at Seoul National University, Suwon, South Korea (37.2°N, 126.9°E). For the light treatments, a greenhouse was divided into three compartments, and the area of each sector was 2.3 W × 9.0 L m. The growing beds were placed in an east-to-west orientation, and the distance between rows was 1.05 m. The nutrient solution was supplied by drip irrigation based on the measured solar radiation inside the greenhouse, and the mean pH and electrical conductivity (EC) values of the solution were controlled at 5.8 and 3.0 dS m^–1^, respectively. When the main stems split into two nodes, each node was pruned with a “V” stem trellis system (Jovicich et al., 2004). The stem density was initially 3.0 stems m^–2^ and increased to 6.0 stems m^–2^ after pruning.

### Measurement of Light Environment

The DLI of natural light was monitored with three sensors on the top of the greenhouse using the quantum sensors (SQ-110, Apogee Instruments, Logan, UT, United States) equipped with a data logger (CR1000, Campbell Scientific, Logan, UT, United States). To analyze the spectral change under the supplementary interlighting, the spectral intensities at the upper, middle, and lower canopies were measured on 2020.11.16∼2020.11.20, four times between 10:00 and 16:00 with a portable spectroradiometer (C-7000, SEKONIC, Nerima-Ku, Tokyo, Japan).

### Light Treatments

**FIGURE 1:**
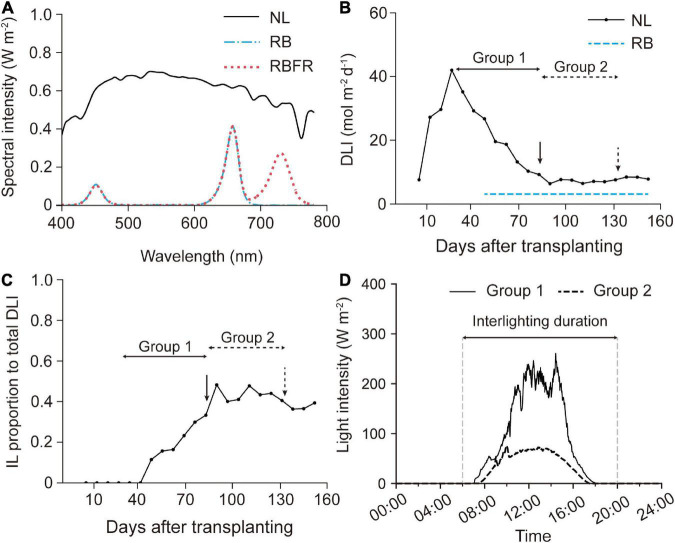
Light environment in the greenhouse during cultivation: spectral composition **(A)**, weekly average of the day light integral (DLI) **(B)**, interlighting (IL) proportion to total DLI (V) **(C)**, and averaged daily light intensity **(D)**. NL, RB, and RBFR mean natural light, NL with red + blue LED interlighting, and RB with far-red light, respectively.

Three light treatments were applied: natural light (NL, control); NL with red + blue LED interlighting (RB); and RB with FR light (RBFR). Two layers of customized interlighting LED fixtures (BISSOL LED, Seoul, South Korea) were installed at heights of 90 and 110 cm above the growing beds. The photosynthetic photon flux density (PPFD) of the single-layer LED fixture was adjusted to 71 μmol m^–2^ s^–1^ at a distance of 20 cm. The red:blue ratio in the RB was 8:2 in PPFD (Figure 1A). Under the RBFR, the additional FR light intensity was set to 55 μmol m^–2^ s^–1^ in photon flux density. Interlighting began on 2020.10.15, after 50 days after transplant (DAT), when the plant heights reached approximately 80 cm when the meristems reached the light sources. All of the interlighting were carried out for 12 h from 6:00 to 18:00 (Figure 1D). Each treatment was blocked with an impermeable plastic film to prevent interference from light sources.

### Fruit Sampling and Color Measurement

**FIGURE 2:**
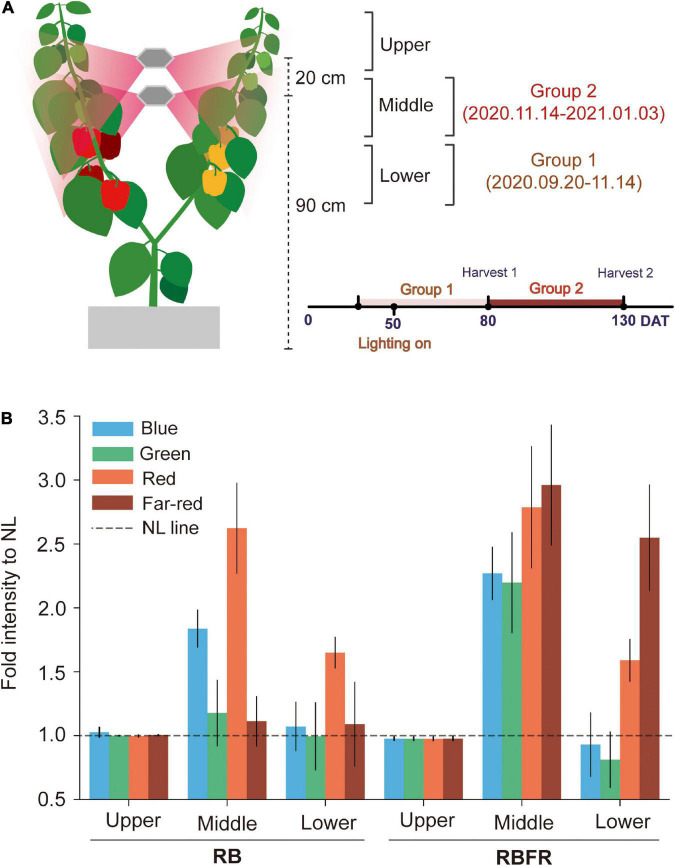
Fruit sampling groups and spectral light distribution in sweet pepper canopy positions. Representation of the fruit harvesting groups and sampling positions **(A)**, and fold intensity of spectral distributions compared to NL from interlighting RB and RBFR measured at the upper, middle, and lower canopy positions **(B)**. NL, RB, and RBFR mean natural light, NL with red + blue LED interlighting, and RB with far-red light, respectively. DAT means days after transplanting, spectral ranges of blue, green, red, and far-red were 400–500, 500–600, 600–700, and 700–750 nm, respectively. Values are the mean ± standard deviation (±SD) of 20 replicates.

Fruit sampling was divided into two groups, Groups 1 and 2 (Figure 2A). Group 1 tracked the fruits that developed from 1 to 3 flowering nodes, and Group 2 was followed by tagging from the beginning of the Group 1 harvest. The fruits in Groups 1 and 2 were monitored with tag tape starting from 25 (2020.09.20) to 80 DAT (2020.11.14), respectively, and ten fruits in each group were monitored. The tagged fruits of Groups 1 and 2 were located about 50–75 and 75–100 cm above the growing bed, respectively (Figure 2A). The L*, a*, and b* color spaces were measured by averaging the measured values obtained with a portable spectrophotometer (CM-2500d, Konica Minolta, Tokyo, Japan) at four random locations on the tagged fruit surfaces. The time when the fruit widths reached 1 cm was regarded as fruit set and determined as 1 day after pollination (DAP). In the tagged fruits, the coloration was measured at 10-day intervals until 30 DAP and at 3-day intervals thereafter. The tagged fruits were harvested at the mature stage when approximately 95% of the fruit surfaces were colored (Verlinden et al., 2005). The harvested fruits were stored in a deep freezer to analyze the fruit qualities and chemical compositions. All analyses were performed in the same manner for the red and yellow fruits.

### Analysis of Fruit Quality

The quality of fully matured fruits was analyzed within 24 h after harvesting. Individual fruit fresh weights were measured with a weighing scale. The fruit dry weights were measured after drying the fruits in a dry oven at 80°C for 72 h. The firmness of fruits was measured with a texture analyzer (CT-3, Brookfield Co., Middleborough, MA, United States). The equatorial planes of the fruits were compressed by using a rounded tip probe of 3-mm diameter at a speed of 10 mms^–1^ and strain of 5 mm. The total soluble sugar (TSS) contents of fruits were measured with a refractometer (PAL-1, Atago, Tokyo, Japan). Titratable acidity (TA) was measured using a fruit acidity meter (GMK-835N, G-WON Hitech Co., Ltd., Seoul, South Korea).

### Quantification of Total Ascorbic Acid

The total ascorbic acid contents were determined with some modifications based on the work of Lee et al. (2017). The reagents, L-ascorbic acid (AA), meta-phosphoric acid (MPA), and dithiothreitol (DTT) were supplied by Sigma-Aldrich (Darmstadt, Germany). For determination of the AA contents, sliced fruits were frozen with liquid nitrogen and stored at −80°C in a deep freezer (DF8520, Dongducheon, Gyeonggi-do, South Korea). Five grams of pulverized frozen sample were mixed with 15 mL of MPA solution. The mixed sample volume was adjusted to 50 mL (5 g/50 mL) and centrifuged for 10 min at 10,000 rpm. Five hundred microliters of supernatant was mixed with 500 μL of DTT solution and incubated for 30 min at 25°C. The aliquots were filtered with a 0.45-μm PVDF membrane filter and injected into a high-performance liquid chromatography (HPLC) (YL 9100, Young Lin, Anyang, Gyeonggi-do, South Korea) with a UV detector set at 252 nm. The separation was carried out using a ZORBAX NH_2_ column (4.6 × 250 mm, Agilent Technologies, Santa Clara, CA, United States) with 30 min of run time. Ten micromoles of ammonium dihydrogen phosphate (pH 2.7) was used as the mobile phase at a 1.0 mL min^–1^ flow rate.

### Quantification of Sucrose, Glucose, and Fructose

Three major free soluble sugars, namely, sucrose, glucose, and fructose, were measured using an HPLC (Dionex UltiMate 3000, Thermo Fisher Scientific, Waltham, MA, United States) equipped with a Shodex RI-101 Detector (Showa Denko, Japan, Tokyo). The glucose reagent was obtained from Junsei Chemical Co., Ltd. (Tokyo, Japan), and the sucrose and fructose reagents were obtained from Sigma-Aldrich (Darmstadt, Germany). Five grams of pulverized frozen sample were mixed with triple distilled water, and the mixed volume was adjusted to 50 mL (5 g/50 ml). Each sample was filtered with a 0.45-μm PVDF membrane filter, and 10 μL of filtrate was injected into the HPLC. The separation was conducted using a Sugar-Pak column (6.5 × 300 mm, Water Corp., Perth, WA, Australia) held at a 70°C oven temperature with 30 min of run time. Ten micromoles of HPLC-grade distilled water was used as the mobile phase at a 0.5 mL min^–1^ flow rate. The HPLC measurement results were used to obtain the total soluble sugar contents by summing the individual sugar contents.

### Quantification of Individual Carotenoid Content

The separation of carotenoids was performed by HPLC (Dionex ultimate 3000, Thermo Fisher Scientific, Waltham, MA, United States) with some modifications based on the work of Yoo et al. (2017). Nine carotenoids were used as standards: capsanthin, capsorubin, zeaxanthin, β-cryptoxanthin, α-carotene, β-carotene, violaxanthin, lutein, and zeaxanthin. All experiments were performed in the dark to prevent carotenoid degradation. First, 100 mg of pulverized frozen samples was homogenized for 1 min with two 6-mm glass beads, 300 μL of tetrahydrofuran (THF), 300 μL of methanol (MeOH) containing 5% butylated hydroxytoluene (BHT), and 50 μL of Mg-carbonate in a 2-mL tube using a TissueLyser II (QIAGEN, Hilden, Germany), and the solution was incubated at 4°C for 20 min. The extract was centrifuged at 4,000 rpm and 4°C for 5 min, and the supernatant was transferred to a new 2-mL tube. Next, the supernatant was mixed with 375 μL of petroleum ether and 150 μL of 25% NaCl, the mixed sample was spun down at 4,000 rpm and 4°C for 3 min, and the supernatant was dispensed into a new 2-mL tube. Then, the sample was dried with a high-speed vacuum centrifuge for 2 h at 45°C. After dispensing 300 μL of 20% KOH + MeOH to the dried sample, shaking incubation was performed at 60 rpm for 10 min. Then, 600 μL of THF, 375 μL of petroleum ether, and 150 μL of 25% NaCl solution were added to the sample, and the supernatant was transferred into a new 2-mL tube by centrifugation for 3 min at 4°C. Supernatant separation was repeated with petroleum ether until the lower phase lost its carotenoid color. The extract was dried using a high-speed vacuum centrifuge for 2 h at 45°C after dispensing 500 μL of acetone into the sample tube, vortexing, and the carotenoid particles were completely dissolved with a sonicator. HPLC was conducted after filtering the samples into amber vials.

### Statistical Analysis

One-way analysis of variance (ANOVA) was conducted using R software version 3.6.1 and the “Agricolae” package. For the *post hoc* test, Tukey’s test (*P* < 0.05) was used when the number of experimental groups was the same, and in other cases, the Bonferroni correction test (*P* < 0.05) was used. All graphics were plotted by using the Python library Matplotlib, version 3.5.1.

## Results

### Light Environment Under the Interlighting

Sweet pepper plants received most of the total PPFD from natural light until the Group 1 fruit harvest (∼80 DAT) (Table 1 and Figure 1B). After the plants nearly reached the interlighting LEDs, operation of the LEDs began (50 DAT). During the Group 1 period, LED lighting was applied for 1 month. The proportion of light that was supplied by interlighting was 13% of that provided by natural light in Group 1. As the amount of natural light decreased in winter (Figure 1B), the proportion of light that was supplied by interlighting increased to 40% of the total PPFD during the Group 2 period (Figure 1C). The average temperature and daytime humidity were maintained at 25°C and 56%, respectively (Supplementary Figure 1). For the spectrum, the fractions of the red, blue, or far-red light increased by 2- to 3-fold in the middle canopy under the RB or RBFR, respectively (Figure 2B). In the lower part of the canopy, the relative proportion of red light increased by 1.7-fold under the RB and RBFR compared to the NL, and the relative proportions of blue light exhibited no significant differences among the light treatments (Figure 2B).

**TABLE 1:** Spectral light intensities (μmol m^–2^ s^–1^) of the light treatments at different growth periods.

Light treatment	Sampling period^z^	PPFD^y^					Average YPFD^w^	R:FR ratio^v^
				
		Blue	Green	Red	Far-red	Total		
NL	Group 1 (2020.09.20-11.14)	64.6	90.5	101.5	47.8	335.9	216.7	2.12
RB		6.2	0.2	36.5	0.0	42.9	38.8	–
RBFR		6.2	0.2	36.7	40.4	43.1	46.4	0.91
NL	Group 2 (2020.11.14-01.03)	19.5	29.3	32.0	15.6	105.6	71.4	2.05
RB		10.3	0.3	60.8	0.0	71.4	64.7	–
RBFR		10.3	0.3	61.0	67.4	71.6	77.4	0.91

*NL, RB, and RBFR mean natural light, NL with red + blue LED interlighting, and RB with far-red light, respectively. The interlighting spectra were measured at a distance of 20 cm.*

*^z^Sampling period indicates the harvesting periods for Groups 1 and 2 referred to in Figure 1.*

*^y^PPFD, photosynthetic photon flux density (μmol m^–2^ s^–1^).*

*^w^YPFD, yield photon flux, normalized ranges of 360–760 nm (McCree, 1971; Sager et al., 1988).*

*^v^R:FR ratio, photon irradiance (666–775 nm)/photon irradiance (725–735 nm) (Franklin, 2008).*

### Fruit Yield and Individual Fresh Weight

The total fruit yields of the red and yellow sweet pepper cultivars were markedly increased under the RBFR compared to the NL and RB (Table 2). In particular, fruit yields under the RBFR were 9 and 19% higher than under the NL for red and yellow fruits, respectively. Under the RB, the individual fruit weights per fruit were significantly lower than those for the NL and RBFR for both fruit colors. For the red fruits, the lengths and widths were shorter under the RB than under the NL and RBFR. For the yellow fruits, only the fruit widths were significantly smaller under the RB than under the NL. There were no significant differences among the treatment groups in the period from the day after pollination (DAP) to harvest (Table 2).

**TABLE 2:** Fruit yield, individual fruit weight, length, and width of harvested red and yellow sweet peppers grown under natural light (NL), NL with red + blue LED interlighting (RB), and RB with additional far-red light (RBFR).

Light treatment	Fruit color	Fruit yield (kg/m^2^)	Individual fruit weight (g/fruit)	Fruit length (cm/fruit)	Fruit width (cm/fruit)	DAP to harvest
NL	Red	1.207	190.9 ± 39.2a*	8.53 ± 1.08a	7.32 ± 0.58a	48.2 ± 8.7
RB		1.477	172.6 ± 43.3b	7.84 ± 1.11b	6.95 ± 0.81b	47.6 ± 4.6
RBFR		1.607	192.8 ± 44.8a	8.35 ± 1.09a	7.28 ± 0.87a	48.0 ± 5.6
NL	Yellow	1.492	207.0 ± 39.0a	8.00 ± 0.83a	7.52 ± 0.59a	52.2 ± 9.5
RB		1.516	194.0 ± 57.4b	7.72 ± 0.96a	7.16 ± 0.74b	51.3 ± 7.4
RBFR		1.806	206.0 ± 49.1a	8.03 ± 1.14a	7.40 ± 0.95ab	50.5 ± 5.7

*Different letters indicate significance among treatments within the same fruit color by using the Bonferroni correction for multiple testing with a significant level of α = 0.05. Mean ± SD (n = 91, 123, 124, 104, 113, and 126 for NL-Red, RB-Red, RBFR-Red, NL-Yellow, RB-Yellow, and RBFR-Yellow, respectively, in order of the treatment). DAP means days after pollination.

### Total Soluble Sugar, Titratable Acidity, Firmness, and Ascorbic Acid Content

**FIGURE 3:**
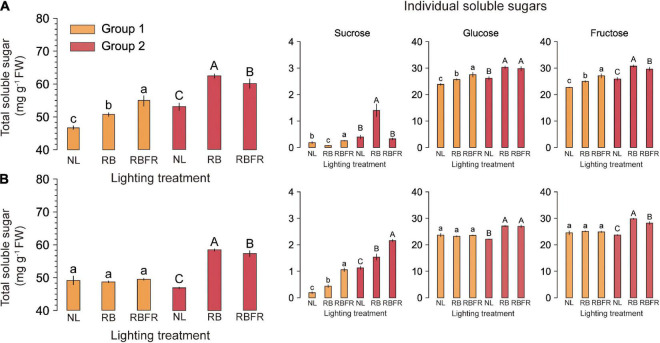
Total and individual soluble sugar concentrations of fully ripened red (A) and yellow (B) fruits of sweet peppers grown under natural light (NL), NL with red + blue LED interlighting (RB), and RB with far-red light (RBFR), respectively. Different letters indicate significance for light treatments within the same fruit color, as determined by Tukey’s HSD test, α = 0.05. Mean ± SD (n = 5). The total soluble sugar was calculated by adding the individual soluble sugars. Groups 1 and 2 indicate fruits sampled at different harvesting periods as shown in Figure 1.

The TSSs of most fruit groups in the interlighting treatments were significantly higher than those under the NL. The RB and RBFR showed significantly higher TSSs in several groups (e.g., 13, 11, 25, 24, and 16% in the order of RB-Red-Group 1, RBFR-Red-Group 1, RB-Yellow-Group 1, RB-Yellow-Group 2, and RBFR-Yellow-Group 2, respectively). The RB showed higher TSSs than the RBFR in the yellow fruit of Group 2. The titratable acidity (TA) showed no significant differences among the light treatments. The TSS:TA was higher under the RBFR than under the NL in the red fruits of Group 1 (Table 3). The ascorbic acid contents exhibited consistent differences among the light treatments and increased with additional FR light levels. Under both the RB and RBFR, the ascorbic acid contents were higher than those under NL, except for the yellow fruit of Group 1. The TSSs estimated from HPLC analysis were higher in the RB and RBFR than under the NL (Figure 3). In the Group 1 samples, only the red fruits exhibited significant differences, which were in the order of RBFR, RB, and NL (Figure 3A). In Group 2, both red and yellow fruits exhibited values that were in the order of RB, RBFR, and NL. The sucrose levels exhibited differences under several light treatments but accounted for 4% of the lower portion in all samples. All of the significant patterns of the glucose and fructose concentrations were consistent with the TSS concentrations except for the glucose concentrations in Group 2.

**TABLE 3:** Total soluble sugar (TSS), titratable acidity (TA), TSS: TA, firmness, and ascorbic acid content of the sampled fruits of sweet peppers grown under natural light (NL), NL with red + blue LED interlighting (RB), and RB with additional far-red light (RBFR).

Light treatment	Fruit color	Sampling period	TSS (brix)	Titratable acidity (g/L)	TSS: TA	Firmness (kg LB/Newton)	Ascorbic acid content (mg/g FW)
NL	Red	Group 1 (2020.9.20-11.14)	6.13 ± 0.25b*	2.64 ± 0.46a	2.37 ± 0.37b	16.2 ± 1.58a	568.8 ± 15.5c
RB			6.90 ± 0.10a	2.79 ± 0.29a	2.5 ± 0.30ab	18.3 ± 2.86a	636.9 ± 7.9b
RBFR			6.83 ± 0.32a	1.92 ± 0.36a	3.63 ± 0.66a	19.3 ± 1.76a	711.0 ± 13.1a
NL	Yellow		6.10 ± 0.30b	2.24 ± 0.18a	2.74 ± 0.27a	14.6 ± 0.69b	722.5 ± 33.8b
RB			7.60 ± 0.17a	2.44 ± 0.43a	3.19 ± 0.64a	18.0 ± 1.33a	729.4 ± 36.0b
RBFR			6.77 ± 0.60ab	1.97 ± 0.22a	3.46 ± 0.33a	16.6 ± 1.53ab	846.4 ± 15.2a
NL	Red	Group 2 (2020.11.14-01.03)	7.90 ± 0.87A	2.93 ± 0.37A	2.70 ± 0.06A	20.6 ± 0.27B	704.9 ± 5.7C
RB			9.87 ± 0.93A	3.37 ± 0.28A	2.93 ± 0.06A	19.9 ± 0.24AB	818.0 ± 10.0B
RBFR			8.16 ± 0.87A	2.35 ± 0.76A	3.61 ± 0.81A	22.3 ± 0.74A	891.7 ± 26.1A
NL	Yellow		7.07 ± 0.23C	3.05 ± 0.47A	2.35 ± 0.31A	13.6 ± 2.02A	767.2 ± 17.0C
RB			8.80 ± 0.08A	3.18 ± 0.61A	2.83 ± 0.53A	15.5 ± 1.70A	1773.3 ± 38.3B
RBFR			8.23 ± 0.31B	3.13 ± 1.10A	2.92 ± 1.20A	18.5 ± 2.92A	1836.3 ± 16.3A

*Different letters indicate significance for light treatments within the same fruit color, as determined by Tukey’s HSD test, α = 0.05. Mean ± SD (n = 5). Statistical tests in Groups 1 and 2 were indicated by lowercase and uppercase letters, respectively. The sampling period indicates the harvesting periods for Groups 1 and 2 referred to in Figure 1.

### Fruit Coloration and Carotenoid Content

**FIGURE 4:**
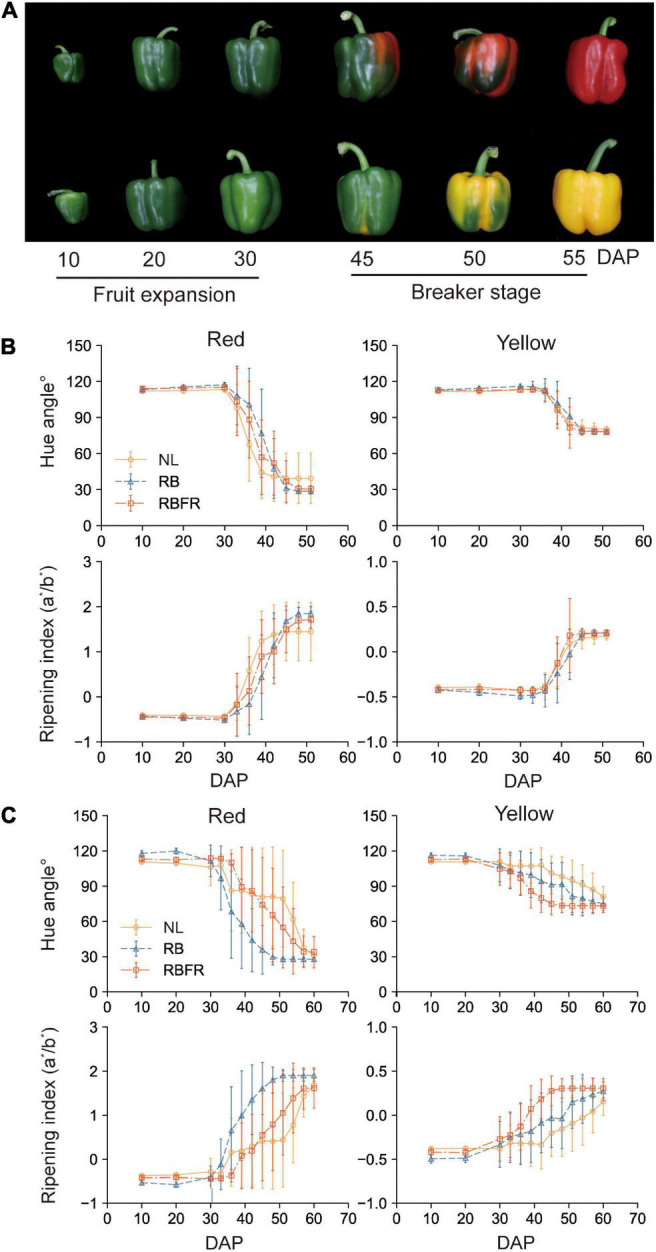
Fruit coloration of red and yellow fruits of sweet peppers grown under natural light (NL), NL with red + blue LED interlighting (RB), and RB with far-red light (RBFR), respectively. Developmental process of sampled fruits **(A)**, hue angles, and ripening index of red and yellow fruits according to DAP in Groups 1 **(B)** and 2 **(C)**. The hue angles and ripening indices were calculated from the measured L*, a*, and b* values. Hue angle was calculated with 180 + tan^–1^ (b*/a*). Groups 1 and 2 indicate fruits sampled at different harvesting periods as shown in Figure 1. Values are the mean ± standard deviation (± SD) of 30 replicates.

**FIGURE 5:**
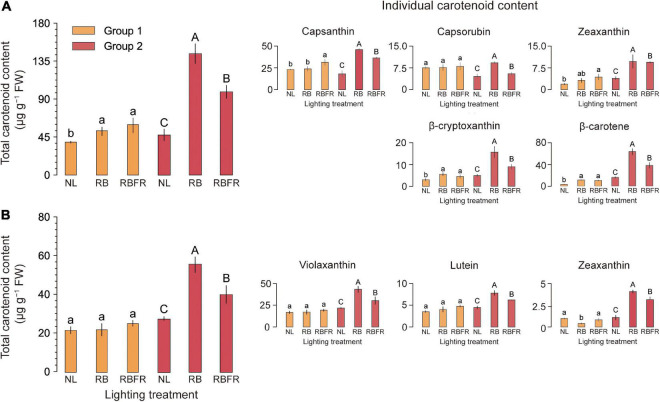
Total and individual carotenoid contents (μg g^–1^ FW) of red **(A)** and yellow **(B)** fruits of sweet peppers grown under natural light (NL), NL with red + blue LED interlighting (RB), and RB with far-red light (RBFR), respectively. Different letters indicate significance for light treatments within the same fruit color, as determined by Tukey’s HSD test, α = 0.05. Mean ± SD (n = 4) Groups 1 and 2 indicate fruits sampled at different harvesting periods as shown in Figure 1.

**FIGURE 6:**
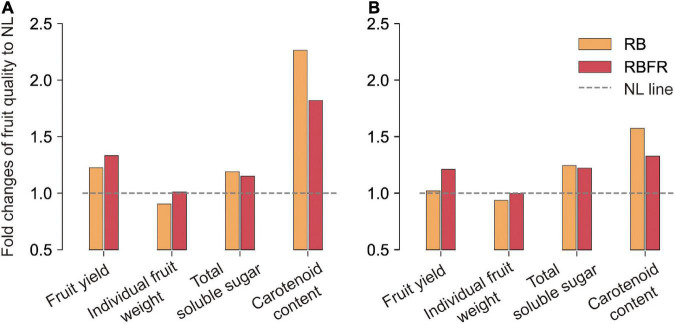
Fold changes in fruit qualities of red **(A)** and yellow **(B)** fruits of sweet peppers to natural light (NL) during overall cultivation period. RB and RBFR mean NL with red + blue LED interlighting and RB with far-red light, respectively. Total soluble sugar and carotenoids content were calculated from data over all sampling periods (Referred to Figures 3, 4).

The surface of sweet pepper fruit color is unevenly ripened (Figure 4A). After breaker stage (DAT 45), the surface color was not significant as it exhibited very large standard deviations across all treatments (Figures 4B,C). There was no trend in fruit ripening rate in Group 1, but in Group 2, fruit ripening was partially promoted at DAT 40 to 50 under RB and RBFR in red and yellow fruits, respectively. Among the nine carotenoids analyzed in this study, eight were detected except for α-carotene, which were capsanthin, capsorubin, zeaxanthin, β-cryptoxanthin, and β-carotene in red fruits and violaxanthin, lutein, and zeaxanthin in yellow fruits (Supplementary Figure 2). In Group 1, only red fruits exhibited significant increases in carotenoid content from the RB and RBFR compared to the NL (Figure 5A), and the total carotenoid contents were not significantly different between the RB and RBFR (Figure 5). In Group 2, the total carotenoid content in red and yellow fruits was higher in the order of RB, RBFR, and NL (Figure 5). The total carotenoid content in Group 2 were 3.0- and 2.1-fold higher than those for the NL in red and yellow fruits in the RB and were 2.0- and 1.4-fold higher under the RBFR, respectively (Figure 5). The individual carotenoid content was significantly lower in red fruits under the RBFR than those under the RB, except for zeaxanthin, and in the yellow fruits, all individual carotenoid content was lower under the RBFR than under the RB. During total sampling period, total carotenoid content in red and yellow fruit was improved compared to NL, about 57 and 33% under the RB and 24 and 22% under the RBFR, respectively. Nevertheless, the RBFR showed approximately 18–24% lower total carotenoid content for both fruit colors than the RB during overall period (Figure 6).

## Discussion

### Interlighting Effects According to the Fruit Vertical Position

Greenhouse crops, such as tomato and cucumber, have trellised training systems that continuously prune while winding the stems into the growing beds between two anchor posts (Hanafi and El-Fadl, 2000). Therefore, the positions of the existing stems continue to move downward during cultivation. On the contrary, in sweet pepper, since it is vertically trellised upward (Jovicich et al., 2004), the shoot meristems of the plants move upward with increasing plant height with growth progress. In this study, since the interlighting LEDs were fixed, the effects of interlighting may vary depending on the vertical positions of the leaves or fruits of the plant. Considering the heterogeneity of the vertical positions of the fruit sets in the canopy, fruit sampling was performed twice during the growing period to equally analyze the fruit qualities in Groups 1 and 2 (Figure 2). In Group 1, since the plants were grown during a period when the amount of natural light was relatively high, the effect of the light spectrum was relatively small (Figure 1C and Table 1). In Group 2, the amount of natural light decreased as winter progressed, and interlighting was conducted in all period after fruit setting (Table 1). In addition to the effect of natural light, the plants had not yet reached the position of the interlighting LEDs, so the lighting durations for Group 1 were relatively shorter for 30 days. Thus, we divided all of the fruit quality analyses into two groups to evaluate the overall effects of interlighting under different natural light backgrounds during total cultivation period.

### Increases in Fruit Yield and Size Under RB With Far-Red Light Interlighting

Several studies have reported increased tomato yields and individual fruit fresh weights under red and blue light with additional FR light (Kalaitzoglou et al., 2019; Kim et al., 2020). Similarly, in this study, greater individual fruit fresh weights and yields were observed under the RBFR than under the RB for both red and yellow fruits (Table 2). The fruit lengths and widths were greater under the RBFR than under the RB, directly related to the higher individual fruit weights and yields. The increases in fruit yield have been explained by the increases in photosynthetic products (Zhen and Bugbee, 2020) or by higher dry matter partitioning to fruits (Ji et al., 2019) induced by FR light. In this study, the RBFR did not change the individual fruit weights compared to the NL, but these decreased under the RB (Table 2). The RB showed much higher red and blue proportions than the NL in the middle canopies (Figure 2B). Therefore, the individual weight losses under the RB may occur due to the spectral effect from higher R:FR or blue light. In the middle or lower canopy, the FR light generated by the natural shading from upper leaves may be insufficient because its intensity depends on the intensity of the solar irradiance. This result suggests that adding FR light to the RB interlighting could improve the yields not under the top lighting but also due to the inter- or intra-canopy lighting in deeper canopies.

### Physicochemical Changes in Fruits Under the Red + Blue LED Interlighting and RB With Far-Red Light Interlighting

Fruit quality factors, such as TSS, TA, and firmness, are essential physicochemical factors that can determine the basic fruit taste or texture of sweet peppers (Ghasemnezhad et al., 2011). To date, there have been studies on the fruit yields under red and blue light in sweet peppers (Joshi et al., 2019; Sobczak et al., 2020), but the effect of FR light on fruit quality under greenhouse conditions has rarely been investigated. In this study, the TSSs were higher under the RB or RBFR than under the NL but did not significantly increase with additional FR light (Table 3 and Figure 3). In tomatoes, when FR light was added to red light, higher TSS levels were reported than when using only red LEDs (Kim et al., 2020). It was also reported that FR light could upregulate sugar transportation and metabolism in tomato fruits by overexpressing those genes related to starch synthases in chamber conditions (Ji et al., 2020). In Group 2, the RB and RBFR increased the individual sugar contents of sucrose, glucose, and fructose compared to the NL (Figure 3), which is consistent with no increase in TSS (Brix°) due to additional FR light (Table 3). This experiment was conducted without shading curtains, unlike previous studies conducted under limited natural irradiance (Ji et al., 2020), since interlighting provides a small portion of the total light. Thus, FR light intensity could not be sufficient to improve the fruit soluble sugar. Reasons other than natural light may be the fruiting properties of sweet peppers. Unlike tomatoes with 3 or 6 fruits hanging per truss (Abdalla and Verkerk, 1968), sweet peppers do not have more than one fruit on each node, so the total number of fruits may vary depending on the light treatment. Since the total number of fruits in this study was lower under the RB, splitting photosynthetic products to each fruit may differ among treatments. Although higher TSSs were observed under both interlighting treatments than under the NL, additional PAR is thought to increase carbohydrate assimilation to the fruit due to the higher photosynthate production in leaves. However, additional FR light did not significantly affect the TA, TSS:TA, or firmness. The ascorbic acid concentrations were higher under the RBFR and RB than under the NL in Group 2. Higher PAR levels resulted in increased ascorbate accumulations in tomato leaves and fruits (Massot et al., 2012; Zushi et al., 2020), similar to our results in sweet pepper fruits. In addition, the ascorbate contents in Group 2 increased by 9 and 4% in red and yellow fruits, respectively, under the RBFR. In previous studies, a decrease in R:FR induced a decrease in ascorbate, but this decrease was consistent with the low level of light irradiance in the natural environment. Ntagkas et al. (2019) reported the effect of exogenous FR light on postharvest tomato fruits, but the effects of the light spectrum during cultivation need to be further studied. In contrast with tomatoes, the ascorbate levels in sweet pepper fruits are highest during the immature green stage and gradually decrease during ripening. Therefore, an individual study of sweet peppers is needed to analyze the effect of light quality.

### Carotenoid Accumulation in Fruits Affected by the RB With Far-Red Light Interlighting

Eight carotenoid components were analyzed to investigate the effect of interlighting on the carotenoid content of red- and yellow-colored fruits. The major carotenoids are capsanthin and capsorubin in red fruits and violaxanthin and lutein in yellow fruits. In plants, the accumulations of plastids in fruit are closely related to light (Llorente et al., 2017). Previous research has revealed increased total carotenoid content under red or blue LED light in controlled environments (Naznin et al., 2019). There have been no reports on the effects of red, blue, and FR interlighting on the carotenoid content of sweet peppers in greenhouse conditions. In this study, the RB showed a 3-fold higher total carotenoid content than the NL in Group 2. However, the total carotenoid content was relatively higher under the RBFR than under the NL by 2.1-fold but was approximately 40% lower than those under the RB (Figure 5). This tendency was consistent for both red and yellow fruits. Despite the equal amounts of PAR light, the RBFR showed a lower total carotenoid content. Llorente et al. (2016) reported that several fruits that accumulate plastids after breakers, such as tomatoes, R:FR, can act as a signal for starting plastid accumulation. We speculated how the fruit carotenoids changed by adding FR to the RB lighting, even in a greenhouse with sunlight background. For the individual carotenoid components, the patterns of significance in the total carotenoid content were equally shown as RB > RBFR > NL in red and yellow fruits, which indicated that the two fruit colors responded similarly to the interlighting. Unlike other studies on tomatoes in which the lighting changed the surface color, no significant differences in fruit color were found (Figures 4B,C). This result seems to be because the color of the sweet pepper surface changes irregularly as fruits ripen (Figure 4A). This study analyzed the qualities of fully ripened fruits, and there was no significant difference in the time it takes to harvest (Table 2). However, practically sweet pepper fruits were harvested when about 80% of the color is colored for shelf-life. During Group 2, overall pigmentation was accelerated by RB and RBFR treatment for about 5–10 days (Figure 4C). The light treatment has the effect of advancing the harvest time like other fruits (Xie et al., 2019). In fruit carotenoids, additional FR light could result in the relatively lower carotenoid than those under RB interlighting alone. This trend was different depending on the harvesting group. In Group 1, no significant differences in total carotenoid content were found in red and yellow fruits, and the levels of only several individual carotenoids increased in red fruits. This result means that the effect of interlighting may also be different depending on the vertical position of the fruit hanging on the stem. Therefore, for the case of crops that are continuously attracted upward with harvesting, such as sweet pepper, it is necessary to move the lighting system upward to where the fruits or leaves are located to obtain the optimal light effects.

### Fruit Qualities Under the Red + Blue LED Interlighting and RB With Far-Red Light Interlighting During Overall Growth Period

The quality of sweet pepper fruits during the total growing period was mostly improved under the RB or RBFR rather than the NL (Figure 6). The fruit yield was higher under the RBFR than the RB, but the carotenoid was higher under the RB than the RBFR. Indices that increased similarly in both treatments were soluble sugars and ascorbic acid (Figure 6 and Table 3). When interlighting was added to NL, the amount of PAR light increased the same, but only the light quality changed. From these results, soluble sugar and ascorbic acid in sweet pepper might react more sensitively to light quantity, and carotenoid and individual fruit weight affected by both light quantity and quality (e.g., R:FR, Blue-far-red interaction) (Brown et al., 1995; Demotes-Mainard et al., 2016). In addition, the effects of such carotenoid improvement may be attenuated by additional far-red light due to antagonism with red or blue light (Llorente et al., 2016; Park and Runkle, 2019). Therefore, it is necessary to choose whether to add far-red light to the interlighting wavelength, focusing on two different purposes: improving yield or fruit functional properties.

## Conclusion

Supplemental lighting with red + blue light (RB) or additional far-red light (RBFR) could increase overall fruit yields and qualities, such as fruit soluble sugar, ascorbic acid, and carotenoid content, in sweet pepper compared to solely natural light conditions. However, fruit yields and individual fresh weights were higher under the RBFR than the RB and vice versa for carotenoid content. This study showed that additional far-red lighting has a trade-off relationship between fruit yields and carotenoid content. Thus, it is necessary to provide an adequate light spectrum according to cultivation purposes, such as improving yield or accumulating plastids in fruits.

## Data Availability Statement

The original contributions presented in this study are included in the article/Supplementary Material, further inquiries can be directed to the corresponding author/s.

## Author Contributions

DK and JS designed the study and prepared the manuscript. DK carried out the cultivation, experiment, data collection, formal analysis, data curation, and writing the original manuscript. JS carried out the conceptualization, investigation, writing the manuscript, and supervision. Both authors contributed to the article and approved the submitted version.

## Conflict of Interest

The authors declare that the research was conducted in the absence of any commercial or financial relationships that could be construed as a potential conflict of interest.

## Publisher’s Note

All claims expressed in this article are solely those of the authors and do not necessarily represent those of their affiliated organizations, or those of the publisher, the editors and the reviewers. Any product that may be evaluated in this article, or claim that may be made by its manufacturer, is not guaranteed or endorsed by the publisher.
