# Supplementary material for: Adding Far-Red to Red, Blue Supplemental Light-Emitting Diode Interlighting Improved Sweet Pepper Yield but Attenuated Carotenoid Content
Source: Front Plant Sci. 2022 Jun 21;13:938199. doi: 10.3389/fpls.2022.938199 (PMC9253827; doi:10.3389/fpls.2022.938199)
Supplement: Supplementary file 1 [file Data_Sheet_1.docx]

Supplementary Materials

# Supplementary Figures


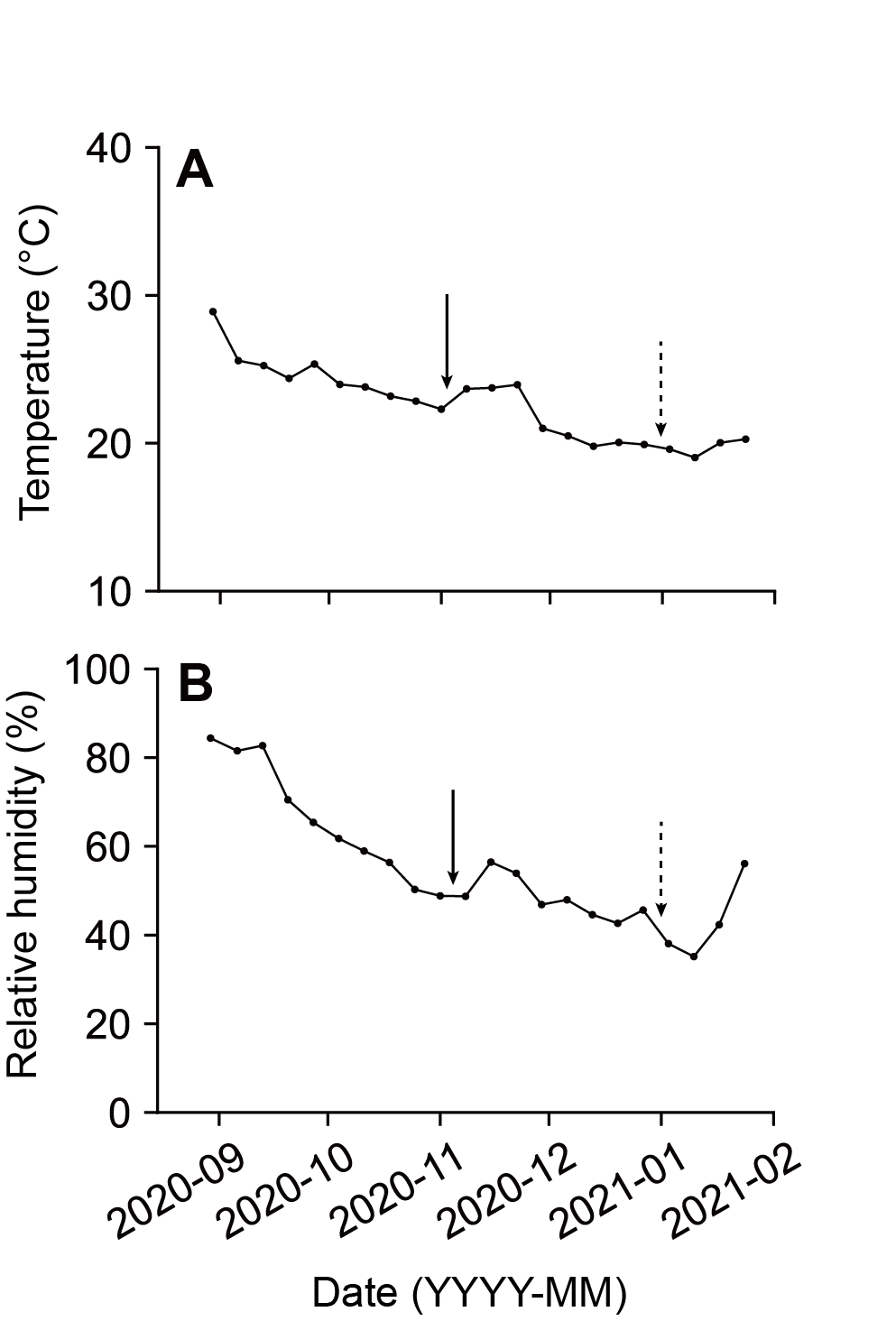


**Supplementary Figure 1.** Temperature (A) and relative humidity (B) in the greenhouse during cultivation.


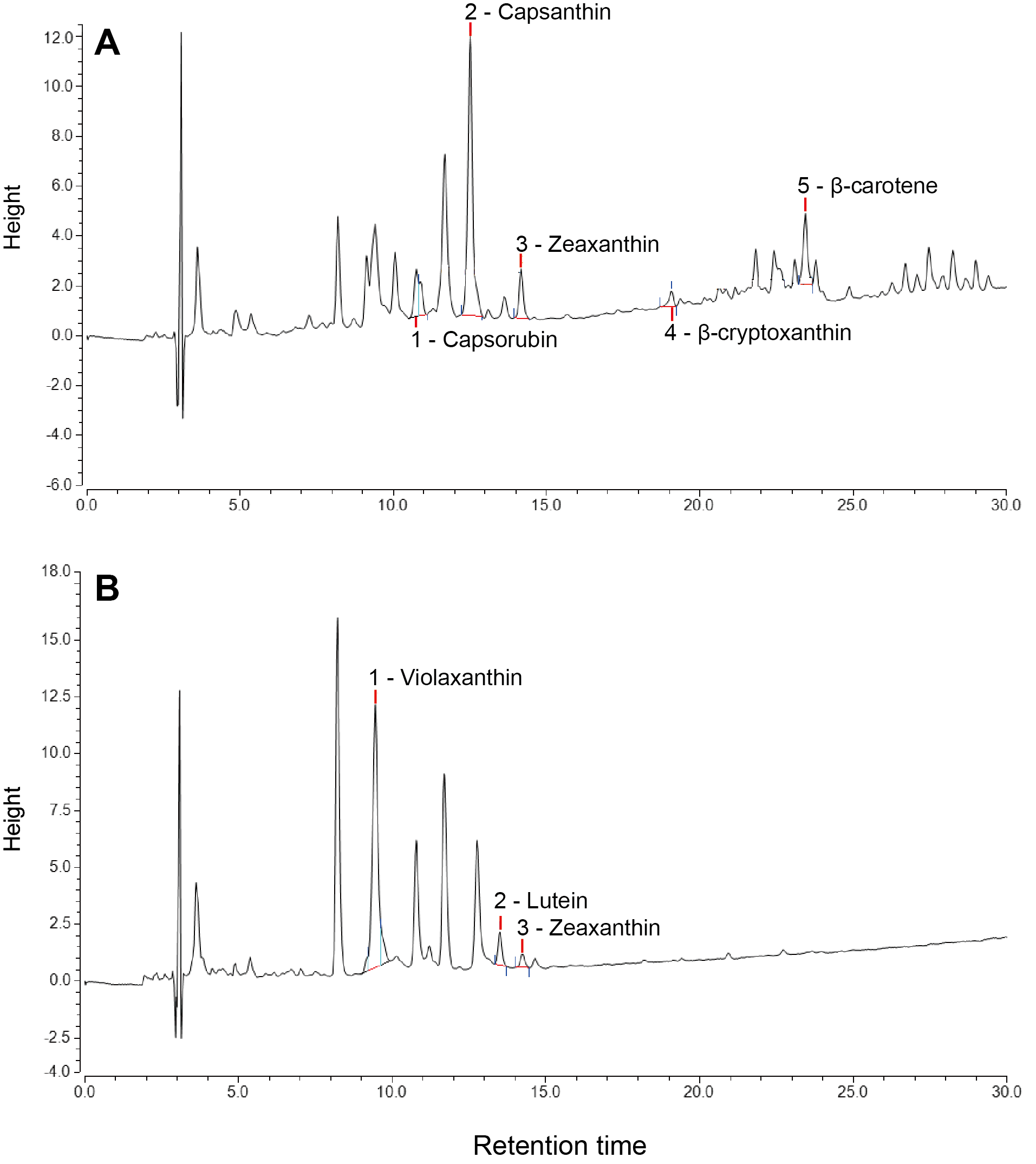


**Supplementary Figure 2.** Chromatograms of the carotenoid profiles in harvested red (“Mavera”, A) and yellow (“Florate”, B) sweet peppers.
